# Curcumin and Related Compounds in Cancer Cells: New Avenues for Old Molecules

**DOI:** 10.3389/fphar.2022.889816

**Published:** 2022-05-24

**Authors:** Matteo Costantino, Cristina Corno, Diego Colombo, Paola Perego

**Affiliations:** ^1^ Unit of Molecular Pharmacology, Department of Applied Research and Technological Development, Fondazione IRCCS Istituto Nazionale dei Tumori, Milan, Italy; ^2^ Department of Medical Biotechnology and Translational Medicine, University of Milano, Milan, Italy

**Keywords:** curcumin, cancer, cell death, proteasome, deubiquitinases

## Abstract

Curcumin and related compounds are known for the large spectrum of activities. The chemical features of these compounds are important for their biological effects with a key role for the thiol-reactive *α*−*β* unsaturated carbonyl groups. Curcumin derivatives may overcome the limitation of the bioavailability of the parent compound, while maintaining the key chemical features responsible for biological activities. Curcumin and related compounds show anti-viral, anti-fungal, anti-microbial and anti-tumor activities. The therapeutic effects of curcumin, used as a supplement in cancer therapy, have been documented in various cancer types, in which inhibition of cell growth and survival pathways, induction of apoptosis and other cell death pathways have been reported. Curcumin-induced apoptosis has been linked both to the intrinsic and extrinsic apoptotic pathways. Necroptosis has also been involved in curcumin-induced toxicity. Among curcumin-induced effects, ferroptosis has also been described. The mechanism of curcumin toxicity can be triggered by reactive oxygen species-mediated endoplasmic reticulum stress. Curcumin targets have been identified in the context of the ubiquitin-proteasome system with evidence of inhibition of the proteasome proteolytic activities and cellular deubiquitinases. Curcumin has recently been shown to act on the tumor microenvironment with effects on cancer-associated fibroblasts and immune cells. The related product caffeic acid phenethyl ester has shown promising preclinical results with an effect on the inflammatory microenvironment. Here, we review the mechanisms underlying curcumin and derivatives toxicity towards cancer cells with particular emphasis on cell death pathways and the ubiquitin-proteasome system.

## Introduction

**FIGURE 1:**
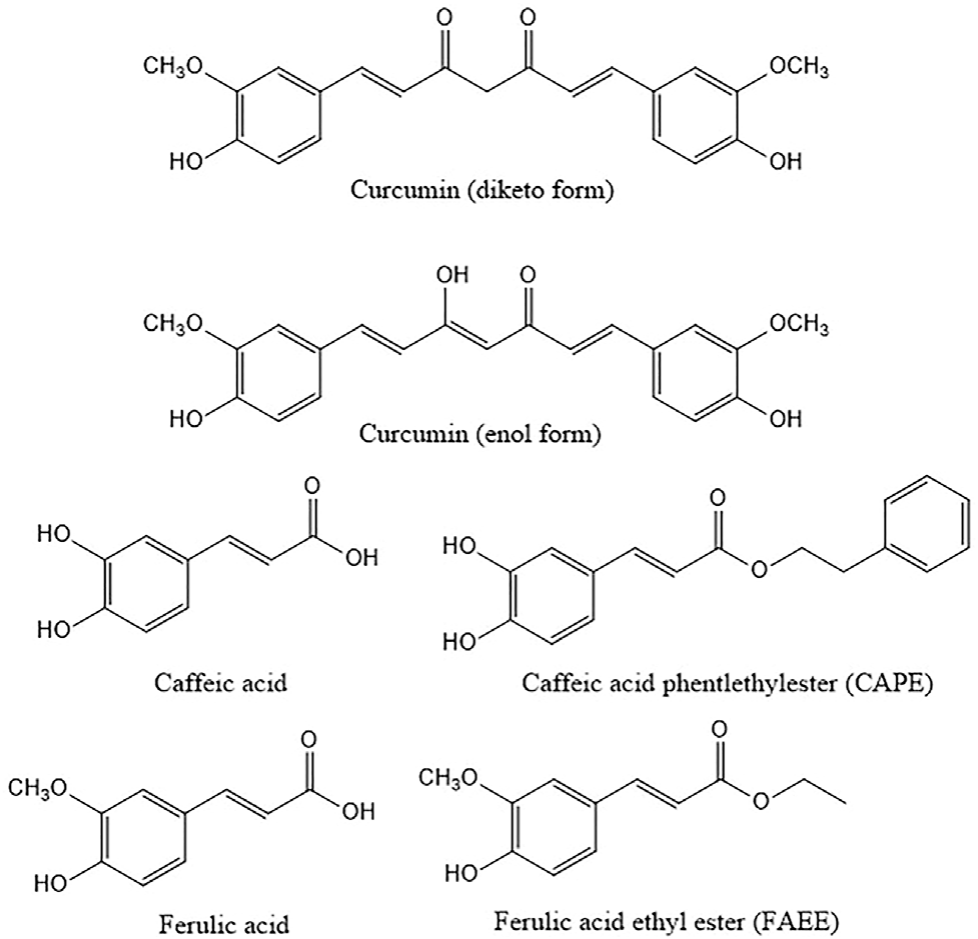
Structures of curcumin and the related compounds caffeic, ferulic acid and their ethyl and phenylethyl esters. Curcumin is here represented in both diketo and enol form the latter being the main form present in solution.

Curcumin and related compounds including caffeic acid phenethyl ester (CAPE), and caffeic acid (Kabala-Dzik et al., 2017) are bioactive compounds mainly derived from natural sources and known for years for their pleiotropic effects (Figure 1). These compounds show anti-viral, anti-fungal, anti-microbial and anti-tumor effects (Chiao et al., 1995; Beauregard et al., 2015; Balaha et al., 2021), mainly ascribable to anti-inflammatory and antioxidant activities. Curcumin product is used as cosmetic additive and dietary supplement and as herbal medicine (Hassanalilou et al., 2019).

Curcumin is a yellow polyphenol component from rhizomes of *Curcuma longa* extracted for the first time in 1815 by Vogel and Pelletier; the structure was identified in 1973 (Kunnumakkara et al., 2017; Mbese et al., 2019). From a chemical point of view, curcumin is a *ß*-diketone. It consists of methane with two hydrogens substituted by feruloyl groups, i.e., dipheruloylmethane. The molecule contains a *ß*-diketone moiety that undergoes keto-enol tautomerization (Nelson et al., 2017). The chemical features of curcumin are important for its biological activity (Giordano and Tommonaro, 2019). Curcumin has been reported to be a pan-assay interference compound (PAINS), that means a compound with possible interference with all assays. Besides, it is an unstable molecule, characterized by reactivity and poor bioavailability. The investigation of stability under physiological and various pH conditions has revealed marked instability (Wang et al., 1997), already in the nineties. Several studies have highlighted the role of the thiol-reactive *α*, *ß* unsaturated carbonyl groups of curcumin and derivatives paying attention to the structure activity relationships (Yang et al., 2017). A key role for the methoxy group has also been proposed (Yang et al., 2017).

The search for new delivery systems for curcumin designed to overcome the low bioavailability, poor water solubility and fast metabolism is an active field of investigation (Wong et al., 2019). Liposomal curcumin has shown marked tumor growth inhibitory activity and proapoptotic effects (Feng et al., 2017). Curcumin solid dispersions have also been reported in an attempt to improve curcumin water solubility (Mohamed et al., 2020). In this context, different hydrophilic carriers have been tested and poloxamer 497 has emerged as promising, allowing curcumin to acquire satisfactory water solubility and dissolution; the complex was more effective than curcumin against colorectal cancer cells (Mohamed et al., 2020). Microemulsions are also being tested as drug carrier to improve curcumin topical delivery (Zainuddin et al., 2021). Of note, curcumin containing micelles have been proposed as a therapeutic supplement in COVID-19 patients (Hassaniazad et al., 2020). Protein/polysaccharide nanocomplexes for delivering curcumin are also being developed; for instance, ovalbumin-pullulan nanogels characterized by improved storage stability have been shown to facilitate controlled release (Zeng et al., 2022). In this regard, the different curcumin nanocarriers (nanocurcumin) can be categorized based on the *ζ* potential and particle size, parameters that may affect the activity in cancer and normal cells with reference to cell death pathways (Shome et al., 2016; Nasery et al., 2020). Besides, Ram and colleague reported a detailed study regarding curcumin-loaded albumin nanoparticles, determining the optimal size for maximum uptake in the target cell (Das et al., 2019). A list of different biopolymer based nanocurcumins reporting their size, *ζ* Potential and their effects in term of stability, bioavailability and activity on different normal and cancer cell lines and animal models is also reported in an exhaustive review by Nasery et al. (Nasery et al., 2020).

The biological activity of curcumin, observed in spite of its low stability, has been ascribed to degradation products measurable *in vivo*, such as ferulic acid (Shen and Ji, 2012). The esters of ferulic acid and of its metabolic precursor caffeic acid, i.e., ferulic acid ethyl ester (FAEE) and CAPE are also endowed with biological activities (Sultana et al., 2005; Olgierd et al., 2021). The latter was described for the first time in the eighties (Grunberger et al., 1988). Similarly to curcumin, CAPE has been widely investigated over the years (Chiao et al., 1995; Olgierd et al., 2021). It is a component of propolis, which is obtained from different plant parts (e.g., the bark of conifers) and carried by honeybees to the hives to be mixed with bee wax and bee salivary enzymes (Chiao et al., 1995). The available evidence suggests that it exerts a selective effect on cancer cells (Sari et al., 2020).

Several curcumin derivatives are reported in the literature (Kabała-Dzik et al., 2017; Chen et al., 2020) as well as CAPE analogs (Beauregard et al., 2015; Balaha et al., 2021). In the present manuscript we review the available knowledge on the mechanism of action of curcumin and related compounds with major emphasis on novel cell death modes and effect on the ubiquitin-proteasome system (UPS).

## Mechanism of Action of Curcumin and Related Compounds

**FIGURE 2:**
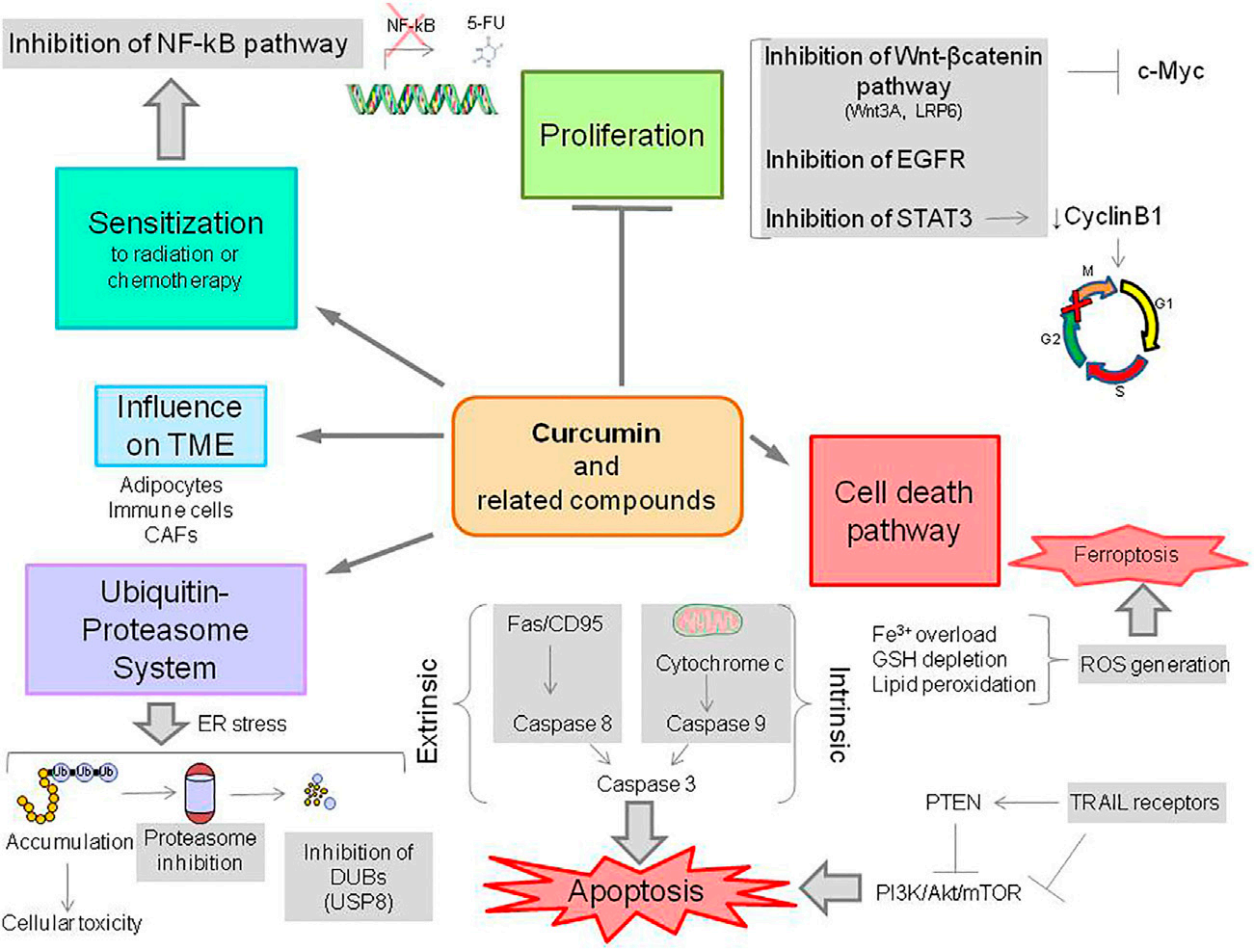
Overview of the biological activities of curcumin and related compounds. CAFs, cancer-associated fibroblasts; DUBs, deubiquitinases; ROS, reactive oxygen species; GSH, reduced glutathione; 5-FU, 5-fluorouracil.

Cell response to stress occurs through different modes including apoptosis. This process which involves an extrinsic and intrinsic pathway provides opportunities to improve the activity of therapeutic agents (Carneiro and El-Deiry, 2020). Indeed, curcumin and related compounds have been regarded as modulators of apoptosis (Reuter et al., 2008) (Figure 2). Recent evidence supports that other less-investigated pathways of cell death, i.e., necroptosis and ferroptosis may play a role in cellular response to curcumin and related compound action (Bertheloot et al., 2021; Lee et al., 2021).

Curcumin and derivatives have been proposed as potential therapeutic agents in selected cancer types such as prostate, colon, breast and thyroid cancer (Schwertheim et al., 2017; Mbese et al., 2019) and their anticancer effects have been tested *in vitro* and *in vivo* in combination with chemotherapeutic agents and radiotherapy (Yu et al., 2021). Curcumin has shown to amplify the anticancer effects of drugs (e.g., doxorubicin, cisplatin, gefitinib) and radiotherapy (Tan and Norhaizan, 2019; Ashrafizadeh et al., 2020). For instance, curcumin displays a synergistic effect with some chemotherapeutic agents such as 5-fluorouracil and oxaliplatin (Farhood et al., 2019) while protecting normal tissues from cell death, and therefore without side effects.

### Inhibition of Cell Growth and Survival Pathways

The action of curcumin and related compounds involves a variety of cellular pathways implicated in key aspects of diseases. Curcumin inhibits the pathway of Nuclear Factor kB (NF-kB), a transcription factor that besides being involved in immune responses and inflammation, acts in regulating genes implicated in cancer development and progression (Dolcet et al., 2005). Curcumin-induced NF-kB inhibition results in sensitization of cancer cells with high expression of NF-kB to radiation or chemotherapy; for example, in cells exposed to 5-fluorouracil induction of high expression of NF-kB has been found in association with resistance to apoptosis and synergism between curcumin and chemotherapy through NF-kB inhibition has been reported for colon and colorectal cancer cell lines (Mortezaee et al., 2019a).

Inhibition of cell proliferation by curcumin has been reported in a variety of cancer cell lines, including non small cell lung cancer cells, in which curcumin-induced ferroptosis (see below) was observed (Tang et al., 2021). In gastric carcinoma cells, curcumin-induced proliferation inhibition was associated with the inhibition of the Wnt/*β*-catenin signaling pathway (Zheng et al., 2017). This pathway plays a crucial role in development, and mutations of Wnt pathway components are linked to growth-related diseases and cancer (Nusse and Clevers, 2017). Curcumin down-regulated Wnt/*β*-catenin signaling by suppressing Wnt3a and low-density lipoprotein receptor-related protein 6 (LRP6) phosphorylation - an event critical for the activation of the pathway - as well as downstream target genes contributing to tumor growth, such as c-myc (Zheng et al., 2017).

Curcumin has been shown to down-regulate Epidermal Growth Factor Receptor (EGFR**)** thereby inhibiting cell proliferation of multiple cancer cells, including lung cancer (Wan Mohd Tajuddin et al., 2019). Of note, a recent *in vitro* and *in silico* study suggests that curcumin and its derivatives are potential tyrosine kinase inhibitors that target EGFR (Liang et al., 2021).

The interference of curcumin with multiple cellular targets appears to underlie its growth inhibitory effect because curcumin has also been shown to decrease eukaryotic initiation factor 2 (eIF2) inhibiting translation and protein synthesis, thereby suppressing proliferation (Chen et al., 2010).

The antitumor effects of curcumin have also been associated to inhibition of Signal Transducer And Activator Of Transcription 3 (STAT3) - which is implicated in sustaining proliferation—resulting in downregulation of cyclin B1 and consequently in G2/M phase arrest in lung cancer cells (Wan Mohd Tajuddin et al., 2019).

Cell cycle-related effects of curcumin have been shown in several studies. For instance, in colon cancer cell lines, curcumin reduced cell growth, with induction of G2/M arrest, and partial G1 phase arrest through the inhibition of cyclin D1, induction of reactive oxygen species (ROS) and down-regulation of E2F4 and other cell cycle related genes (cyclin A, p21, p27) (Pricci et al., 2020).

### Activation of Apoptosis and Other Cell Death Pathways

The contribution of the extrinsic and intrinsic apoptotic pathways to curcumin-induced apoptosis has been recognized in different cellular models. Activation of extrinsic or intrinsic pathways are not necessarily mutually exclusive and they may be both activated by curcumin in some cell types. The predominant apoptotic pathway targeted by curcumin may differ between cell type, differentiation stage or curcumin concentration. Curcumin has been shown to regulate the expression of TRAIL receptors, a phenomenon associated with regulation of phosphatase and tensin homolog (PTEN) and inhibition of PI3K/Akt resulting in apoptosis (Mortezaee et al., 2019b). In gastric cancer cell lines, curcumin exerts apoptotic effects through activation of the intrinsic apoptotic pathway promoting the release of cytochrome c from the mitochondria to the cytoplasm, leading to Bcl-2 decrease, Bax increase, caspase-9 and caspase-3 activation and Poly (ADP-ribose) polymerase (PARP) cleavage (Hassanalilou et al., 2019).

Induction of the intrinsic and extrinsic apoptosis pathways by curcumin occur *via* suppression of COX2 through deregulation of NF-kB, downregulation of Bcl-2 and upregulation of Bax and Bad proteins in lung cancer cells; suppression of the PI3K/Akt/mTOR pathway and enhanced Fas/CD95 expression and caspase-8 activity (extrinsic apoptotic pathway activation) have been observed (Wan Mohd Tajuddin et al., 2019). Curcumin-induced apoptosis has also been related to accumulation of ceramide in the membrane as a consequence of enhanced ROS production in human colon cancer cells (Mortezaee et al., 2019a).

Curcumin induces pro-apoptotic proteins while reducing antiapoptotic proteins in cancer cells, also promotes ROS production to induce apoptosis. Curcumin can also trigger endoplasmic reticulum (ER) stress response by inhibiting proteasomal activity, accumulating cytosolic Ca^2+^, and disrupting disulfide bonds (Reuter et al., 2008).

Besides apoptosis cells can activate additional types of cell death. Ferroptosis is a form of programmed cell death characterized by lipid peroxidation and is inducible by iron and the accumulation of ROS (Jiang et al., 2021). The available evidence suggests that curcumin action is at least in part mediated by ferroptosis. Indeed, in curcumin/curcumin analog-treated cells iron overload was observed together with reduced glutathione depletion and lipid peroxidation (Li et al., 2020; Lin et al., 2021). The phenomenon has been described to be associated with curcumin tumor growth inhibitory effect in various cancer types such as glioblastoma and lung cancer (Chen et al., 2020; Tang et al., 2021). The ferroptotic effect induced by curcumin seems to be direct or indirect, e.g. mediated by excessive authophagy (Tang et al., 2021). A protective effect of curcumin against iron toxicity and Erastin-induced ferroptosis was observed in murine MIN5 pancreatic beta cells., likely dependent on the modulation of the expression and activity of iron metabolism factors such as iron regulatory proteins (IRPs) and transferring receptor one mRNA (TfR1); cotreatment of curcumin and erastin reversed the cell death triggered by erastin and suppressed iron accumulation (Kose et al., 2019).

A cytotoxic effect has also been referred for nanocurcumin, which in general appears to be more potent than curcumin (Table 1).

**TABLE 1:** Cyototoxicity of curcumin and nanocurcumin^a^.

Cell Lines	Curcumin IC_50_	Nanocurcumin IC_50_	References
Breast cancer cells (MDA-MB231)	79.58 μg/ml (24 h) 53.18 μg/ml (48 h) 30.78 μg/ml (72 h)	37.75 μg/ml (24 h) 23.25 μg/ml (48 h) 12.99 μg/ml (72 h)	Khosropanah et al. (2016)
MDA-MB231	n.d	79.58 μg/ml (48 h)	Kumar et al. (2021)
Cervical cancer (HeLa)	50 μM (48 h)	15 μM (48 h)	Sadeghi et al. (2021)
Fibroblasts	>140 μM (48 h)	160 μM (48 h)	Sadeghi et al. (2021)
Pancreatic cancer cell lines (BxPC3, ASPC-1, PL-11 and XPA-1)	n. d	10–15 μM (48 h)	Bisht et al. (2007)
Glioblastoma (U87MG)	20–40 μM	n.d	Chen et al. (2020)
Laryngeal cancer (Hep-2)	n.d	45.1 μg/ml (24 h) 17 μg/ml (48 h)	Demiana and Gamal (2020)
Baby hamster kidney normal cell lines	n.d	>300 μg/ml (48 h)	Demiana and Gamal (2020)

a The cytotoxic effect of curcumin and nanocurcumin is reported as published in the cited references. Exposure times are indicated in brackets. n.d., not determined.

Regarding curcumin capability to activate cell death, the engagement of various pathways has been recently reported (Yu et al., 2021). Besides, in the literature it is widely reported that normal cells are less sensitive to curcumin treatment than cancer cells and curcumin does not induce any apoptotic death in normal cells and tissues (Syng-Ai et al., 2004). However, under selected conditions curcumin may induce cell death in normal cells in dose-dependent manner. For instance, in human osteoblast cells, treatment with curcumin (12.5–25 µM) can activate apoptosis with activation of caspase-3, c-Jun NH2-terminal Kinase (JNK) and PARP cleavage, while treatment with curcumin at higher concentrations (50–200 µM) triggers necrotic cell death. In fact, curcumin affects intracellular ATP pool and ROS generation, involved in the switching between apoptosis and necrosis. (Chan et al., 2006).

The different effects of curcumin in normal cells or cancer cells have not been fully clarified, but some reasons have been suggested, such as a higher uptake and lower glutathione levels in tumor cells as compared to normal cells as well as costitutive NF-kB activation in most tumor cells (Syng-Ai et al., 2004; Shishodia et al., 2005; Kunwar et al., 2008).

### Inhibition of the Ubiquitin-Proteasome System

The UPS consists of enzymes that conjugate ubiquitin (Ub) to/from target proteins upstream of the 26S proteasome. Ub can be conjugated to target proteins as a monomer or as an Ub chain variable in length and type. Lys48-linked Ub chains target proteins to proteasomal degradation, which occurs after an ATP-dependent unfolding of the poly-ubiquitinated substrate that is translocated into the 20S catalytic core, thus becoming available to proteases. The conjugation of Ub is a controlled event involving Ub-activating, -conjugating and ligating enzymes. The reverse reaction is highly regulated and is mediated by a class of enzymes known as deubiquitinases (DUBs) that remove Ub from target proteins (Clague et al., 2019). Cancer cells display high rate of protein synthesis and depend on the UPS for their homeostasis. Indeed, protein degradation participates in the regulation of multiple functions (e.g., signal transduction, transcription, cell cycle) by controlling regulatory protein levels and prevents the accumulation of damaged/misfolded proteins in the cells. Three DUBs are proteasome-associated and their inhibition results in accumulation of poly-ubiquitinated proteins and cell toxicity (D'Arcy et al., 2011). Conversely, several non proteasome-associated DUBs remove Ub from cell survival, DNA damage repair- and apoptosis-related proteins (Clague et al., 2019).

Several reports have provided evidence that curcumin acts as a proteasome inhibitor. Indeed, inhibition of proteasomal function by curcumin has been associated with apoptosis induction through the mitochondrial pathway (Jana et al., 2004). Curcumin inhibited different enzymatic proteasomal activities such as chymotrypsin, trypsin, and post-glutamyl peptidyl-like protease activity, both in murine neuroblastoma and human cancer cells (Jana et al., 2004). Induction of stress response by curcumin has also been related to inhibition of proteasome, preventing NF-kB activation (Dikshit et al., 2006). *In silico* docking studies and nucleophilic susceptibility assays have shown that the carbonyl carbons of curcumin undergoes nucleophilc attach by the hydroxyl group of the N terminal threonine of the chymothrypsin-like subunit of the proteasome, thereby providing a mechanistic interpretation of the proteasome inhibitory action (Milacic et al., 2008). Consistently, curcumin treatment of colon cancer cells results in proteasome inhibition, accumulation of ubiquitinated proteins and induction of apoptosis, features also observed in tumors from xenografted mice (Milacic et al., 2008).

Besides inhibition of the 20S proteasome proteolytic activity, curcumin has been shown to inhibit cellular DUBs (Si et al., 2007), suggesting an impact of this compound on various components of the UPS. Indeed, a pharmacophore model has been proposed according to which curcumin beta-carbon may inhibit ubiquitin-isopeptidase activity (Mullally and Fitzpatrick, 2002). The chemical requirements for inhibition of DUBs are shared by curcumin and most related compounds including CAPE. In fact, a recent study support that CAPE can inihibit the DUB ubiquitin-specific protease 8, but not proteasome associated DUBs in cell-free assays, suggesting partial selectivity (Colombo et al., 2022). CAPE, similarly to what observed for curcumin, has also been shown to reduce 26S proteasome proteolytic activities, mainly affecting chymotrypsin-like activity (Matsunaga et al., 2019). In addition, the interference of curcumin with the proteasome activity has also been linked to a direct inhibition of dual-specificity tyrosine-regulated kinase two which phosphorylates the RPT3 subunit (Banerjee et al., 2018) implying the multimodal nature of proteasome inhibition by curcumin.

### Effects on Tumor Microenvironment

The tumor microenvironment (TME) contributes to response to antitumor therapy. Indeed, the role of key players present in the tumor stroma such as fibroblasts, immune cells, the extracellular matrix and extracellular molecules has been widely investigated leading to the evidence that all these components participate in modulation of tumor cell response to treatment (Wu and Dai, 2017).

The antitumor effects of curcumin have been defined in a large number of *in vitro* and *in vivo* studies, mainly addressing curcumin direct activity on tumor cells with less attention to the TME. A recent study has highlighted the effects of curcumin on prostate cancer-associated fibroblasts (CAFs) in which a reduction of mitochondrial membrane potential and the promotion of the intrinsic apoptotic pathway was observed, together with cell cycle arrest (Zeng et al., 2020). Curcumin pro-apoptotic effect appeared to occur via ROS generation and ER stress involving the ROS-dependent RNA-dependent protein kinase -like ER kinase (PERK)-eIF2α-ATF4 axis (Zeng et al., 2020).

An action of curcumin on adipocytes has been reported. The treatment of preadipocytes with curcumin has a dual effect (Wu et al., 2019). Whereas a high concentration of curcumin induces apoptosis through the activation of both intrinsic and extrinsic apoptosis, a low concentration inhibits adipocyte differentiation through inhibition of cell cycle regulators and down-regulation of adipogenic transcription factors (e.g., PPAR*γ*) (Wu et al., 2019).

Curcumin effects on immune cells have been described, with curcumin ability to regulate immune responses reducing apoptosis in natural killer cells and increasing their cytotoxic activity against cancer cells (Fiala, 2015). In addition, curcumin can potentiate cytotoxic T Lymphocites action against cancer cells and induce polarization of tumor associated macrophages towards inflammatory macrophages, thereby regulating the TME in favor of antitumor immunity (Bhattacharyya et al., 2010; Mukherjee et al., 2018).

The capability of curcumin to interfere with the microenvironment is supported by a study in which curcumin antioxidant action improves stem cell transplantation efficiency. The excess of ROS can damage the mitochondria changing the permeability with release of proapoptotic factors in the cytosol; ROS can also promote lipid peroxidation, increasing levels of malondialdehyde thereby damaging the cell membrane. Curcumin can modulate the expression of oxidative stress regulatory proteins such as SOD2 and GPX1 decreasing intracellular ROS levels (Tan et al., 2021), thereby protecting human periodontal ligament stem cells mitochondria from damage and apoptosis by reducing ROS levels and stimulating the ERK pathway (Tan et al., 2021).

Finally, curcumin related compounds such as CAPE appear also to be endowed with the capability to interfere with the TME as reported in a study showing CAPE inhibition of breast cancer cell proliferation in inflammatory microenvironment associated with activation of apoptosis and inhibition of the TLR4 signaling pathway (Chang et al., 2017).

## Conclusion and Perspectives

Curcumin has been widely studied as anti-aging and chemopreventive agent (Pricci et al., 2020; Zia et al., 2021). Overall, the efficacy of curcumin has been ascribed to the modulation of signaling pathways associated with tumor development and progression via interaction with a variety of proteins involved in proliferation, apoptosis, and inflammation. Opposite effects are described depending on the context (e.g., normal versus tumor cells) (Tan et al., 2021).

A very promising aspect of research on curcumin appears the investigation of strategies to improve the molecule drawbacks or the use of alternative related compounds such as CAPE which seems to be more stable. In this context, the effects of curcumin related compounds on the TME and not only on tumor cells appears attractable. The bioavailability of curcumin, which *per se* cannot be considered a drug in cancer therapy, but a supplement, can be improved by encapsulation in micelles as well as in other types of nanoparticles (Tagde et al., 2021). In this context, the toxicity of surfactants used for such formulations has to be taken into account.

The identification of novel targets for curcumin and related compounds in the UPS and the elucidation of the precise mechanisms of interaction with the proteasome open new avenues to the understanding of the tumor-related effects of these compounds.
